# Impact and Prevalence of Depression and Anxiety in Rheumatoid Arthritis—A Cross-Sectional Study with Self-Reported Questionnaires

**DOI:** 10.3390/jcm14051718

**Published:** 2025-03-04

**Authors:** Cătălina-Elena Ionescu, Claudiu C. Popescu, Cătălin Codreanu

**Affiliations:** 1 Rheumatology Department, “Carol Davila” University of Medicine and Pharmacy, 020021 Bucharest, Romania; catalina-elena.ionescu@drd.umfcd.ro (C.-E.I.); catalin.codreanu@reumatologiedrstoia.ro (C.C.); 2 “Dr. Ion Stoia” Clinical Center of Rheumatic Diseases, 020983 Bucharest, Romania

**Keywords:** rheumatoid arthritis, depression, anxiety, disease activity, quality of life

## Abstract

**Objective:** This study aims to screen for depression and anxiety in a real-life sample of rheumatoid arthritis (RA) patients and to observe whether RA phenotype characteristics and RA disease activity measures are associated with depression and anxiety. **Methods:** This cross-sectional study from a tertiary rheumatology hospital in Romania screened all patients with diagnosed RA that came for their one month disease follow-up for depression and anxiety using the Patient Health Questionnaire-9 (PHQ9) and Hospital Anxiety and Depression Scale (HADS), self-reported questionnaires. The follow-up captured the date of RA diagnosis, pharmacological treatment, clinical examination, blood sampling, and functional and radiographic assessment. The cut-off for positive screening of depression was a PHQ9 of 10 or more and a HADS-depression (D) of over 10, and the positive cut-off for anxiety was a HADS-anxiety (A) of over 10. **Results:** According to the medical histories, the prevalence of depression and anxiety in the 209 patients included was 10% and 8.1%, respectively, while the likely depression diagnosis according to PHQ was 34.4% and that according to HADS-D was 14.8%, while the likely anxiety diagnosis using the HADS-A was 32.5%. The subgroup of patients that positively screened for depression using the self-reported questionnaires PHQ9 and HADS-D had significantly higher DAS28, disease activity class, tender joint count, swollen joint count, patient global assessment, and functional stage, with some particularities regarding ESR and radiographic stage, which were higher just in the HADS-D of more than 10 subgroup, and glucocorticoid use, which was higher just in the PHQ9 over 10 subgroup. Regarding patients with a HADS-A of more than 10, they were more frequently women and had higher tender joint count and functional stage. **Conclusions:** Depression and anxiety are highly prevalent and underreported in the RA population and are associated with higher levels of pain, physical disability, and disease activity.

## 1. Introduction

Rheumatoid arthritis (RA) is a chronic systemic inflammatory disease that involves the synovial joints, where it determines a chronic inflammatory process that leads to early, progressive, and irreversible osteoarticular damage. RA is frequently associated with various extraarticular manifestations, which, through different complications, reduce life expectancy by 5 to 10 years. The prevalence of RA in the general population is 0.5–1%, making it the most frequent rheumatic inflammatory disease, representing a major public health problem [1]. One of the most frequently associated comorbidities in RA is depression, with a prevalence of 15% according to a paper published by Dougados et al. in 2014 [2]. The prevalence of depression has been shown to be 2–3 times higher than in the general population, with a meta-analysis conducted by Matcham et al. in 2013 reporting that 16.8% of RA patients have a major depressive disorder [3]. In different studies, a high variability for depression prevalence in RA has been shown, ranging from 2% in Morocco to 33% in the USA, probably caused by the differences in study design, the variations in the definitions of depression, and the methods used for measuring depressive symptoms [4]. In the general population, depression has a prevalence of 6% [5]. Another frequent comorbidity in RA is anxiety, with 25.1% of patients screening positive according to the Generalized Anxiety Disorder questionnaire [6,7], while in the general population, the prevalence is 4.03% [8]. Both depression and anxiety are screened positive in 16.3% of RA patients [7].

RA patients with associated depression have a poor long-term outcome; they suffer from higher levels of pain, fatigue, disease activity, and physical disability. Depression lowers treatment adherence, causing more comorbidities and leading to higher mortality rates, partly through increased suicide risk. Health service utilization and healthcare costs are increased in these patients directly through hospitalization but also indirectly through loss of work productivity. Ultimately, RA patients with depression tend to have a lower quality of life, a fact that is reflected in all the domains of the Quality of Life questionnaire [9,10].

Associated anxiety worsens several outcomes in RA patients, leading to poor long-term outcome by increasing morbidity, mortality, and pain [11]. It alters sleep patterns, leading to sleep disorders such as early awakening and insomnia. Similarly to depression, anxiety lowers patients’ adherence to treatment, leads to high medical costs through high utilization of health resources [12], increases sick leaves, affecting work productivity [13]. The multiple effects of anxiety on RA patients finally contribute to a decline in their quality of life [14].

The psychiatric interview and diagnosis according to Diagnostic and Statistical Manual (DSM) or International Classification of Diseases (ICD) criteria are the gold standard for diagnosing depression and anxiety [15], but they are expensive and time-consuming, making them difficult to use in a busy hospital setting. In research studies on the prevalence of depression or anxiety in RA, self-reported screening questionnaires are used because they require less funds and are faster. This may lead to overestimation of prevalence, as screening tools tend to prioritize sensitivity over specificity [16].

In 2013, a meta-analysis carried out by Matcham et al. on depression definitions in RA patients showed that some of the screening tools used to detect depression are the Patient Health Questionnaire-9 (PHQ9), Hospital Anxiety and Depression Scale (HADS), Inventory to Diagnose Depression (IDD), Center for Epidemiological Studies Depression Scale (CESD), Beck Depression Inventory (BDI), and the Geriatric Depression Scale (GDS), the most frequently used being the HADS and the CESD. Also, the HADS was the tool most frequently used to identify anxiety. The definition of depression and anxiety based on these questionnaires included possible or probable positive diagnosis by using either one or multiple thresholds [3].

Self-reported questionnaires are not completely accurate, but they are useful tools for screening depression and anxiety in RA patients who need further psychiatric evaluation. Screening for mental illness is extremely important because the growing evidence clearly suggests that detecting and correctly treating depression and anxiety in RA patients leads to a better quality of life by improving pain, fatigue, and physical disability and lowering RA disease activity. The many studies on RA cohorts from different countries that assessed the prevalence of depression and also the relationship between the presence of depression and RA disease activity have different designs and utilize different screening tools with variable thresholds for screening depression as well as multiple depression definitions, which generates high variability of the reported prevalence from 2% to almost 80%. Additional studies could potently contribute to generating an international standardized method for assessing depression in RA patients. Also, socio-economic and socio-cultural factors exhibit a significant impact on the variability of depression prevalence, so we considered it useful to conduct this type of study in our country, hoping to raise awareness about the impact of mental health on disease activity. In this context, the study aims to screen for depression and anxiety a real-life sample of RA patients from Romania and to observe whether RA phenotype characteristics and disease activity measures like Disease Activity Score 28 (DAS28) and its components are associated with depression and anxiety.

## 2. Materials and Methods

### 2.1. Patients

The study screened all patients diagnosed with RA according to their attending physicians who randomly came for follow-up evaluations in the “Dr. Ion Stoia” Clinical Center of Rheumatic Diseases from Bucharest, Romania, between September and November 2024. Additional inclusion criteria were fulfillment of the 2010 American College of Rheumatology—European League against Rheumatism classification criteria for RA [17] and age above 18 years. Exclusion criteria were overlapping chronic autoimmune or inflammatory conditions, a current/recent diagnosis of cancer, other psychiatric disorders (schizophrenia, bipolar disorder, psychosis), experimental RA treatments, substance use disorders, cognitive impairment, pregnancy, and recent major life events (trauma, bereavement). Preexisting depression and anxiety were defined as either patient-reported medical history or if the patients were taking specific psychiatric medication for anxiety or antidepressants (namely tricyclic antidepressants, atypical antidepressants, selective serotonin re-uptake inhibitors, selective serotonin and norepinephrine re-uptake inhibitors, norepinephrine and dopamine reuptake inhibitors, noradrenaline and specific serotonergic antidepressants, serotonin modulators and stimulators, serotonin antagonists and reuptake inhibitors, or monoamine oxidase inhibitors, all prescribed by their treating psychiatrists). All patients gave written informed consent, and the study was approved by the hospital’s ethics committee. Each patient underwent all study procedures in the same day (clinical interview, clinical examination, peripheral venous blood sampling for laboratory tests, hand and feet X-rays, and questionnaire answering).

### 2.2. RA Evaluation

Clinical interviews and/or medical records allowed us to capture the date of RA diagnosis (used to calculate RA disease durations) and current pharmacological treatment (biologic, conventional synthetic, or targeted synthetic disease-modifying antirheumatic drugs—b/cs/tsDMARDs and glucocorticoids). Laboratory tests included autoantibodies (rheumatoid factor—RF and anti-citrullinated protein antibodies—ACPA, if not tested within the last year) and acute phase reactants (C-reactive protein—CRP, normal below 5 mg/L; erythrocyte sedimentation rate—ESR, normal below 20 mm/h), all conducted by the local laboratory using commercially available kits. Each attending rheumatologist prompted the patients to report a global health evaluation (on a 100 mm visual analogue scale—VAS), evaluated disease activity by counting the numbers of tender (TJC28) and swollen joints (SJC28), and classified the patient functionally, using Steinbrocker functional capacity classes [18]. Disease activity classes were defined with DAS28 [19,20], which was calculated with 4 variables with CRP, as follows: remission (DAS28 < 2.6), low (2.6 ≤ DAS28 ≤ 3.2), moderate (3.2 < DAS28 ≤ 5.1), and high disease activity (DAS28 > 5.1). All radiographic progression staging was labeled using Steinbrocker criteria by the clinic’s imaging expert [18].

### 2.3. Depression Questionnaires

Each patient filled in, without external aid, two questionnaires: PHQ-9 [21] and HADS [22]. PHQ9 is a widely used and validated tool for screening, diagnosing, monitoring, and measuring the severity of depression. It consists of 9 items, each corresponding to the diagnostic criteria for major depressive disorder. Questions assess symptoms like mood, sleep, energy, appetite, and suicidal thoughts over the past two weeks. Each item is scored on a scale from 0 (not at all) to 3 (nearly every day), with a total score range of 0–27, with the following categories: minimal depression (PHQ9 = 0–4), mild depression (PHQ9 = 5–9), moderate depression (PHQ9 = 10–14), moderately severe depression (PHQ9 = 15–19), and severe depression (PHQ9 = 20–27). HADS is a validated self-report questionnaire used to assess symptoms of anxiety and depression, which allows for the exclusion of somatic symptoms to avoid confounding with physical illness, making it particularly useful in hospital or outpatient settings. It contains 14 items divided into two subscales for anxiety (HADS-A, 7 items) and depression (HADS-D, 7 items). Each item is scored from 0 to 3, with subscale scores ranging from 0–21. The global score indicate severity: normal (HADS = 0–7), borderline abnormal (HADS = 8–10), and abnormal (HADS = 11–21, clinical level).

### 2.4. Statistics

The normality of data distribution was assessed using descriptive statistics, normality, stem plots and leaves, and Kolmogorov–Smirnov tests corrected by Lilliefors. Continuous variables are reported as mean (standard deviation) if normally distributed, or as median (interquartile range) if non-normally distributed, while dichotomous variables are reported as percentage of group or subgroup. Independent 2-sided t tests or Mann–Whitney U or Kruskal–Wallis tests were used to evaluate differences in continuous variables between subgroups of categorical variables, while associations between categorical variables were studied using χ^2^ tests. Statistical tests were considered significant if *p* < 0.035 for an alpha level of 0.05, and Bonferroni correction was used in order to avoid the type I error. Statistical analysis was performed using IBM SPSS Statistics version 25.0 for Windows (IBM Corp., Armonk, New York, NY, USA).

## 3. Results

The study included 209 patients, of which 92.3% were women, with an average age of 62.1 (11.5) years (Table 1). Most patients had established disease, with a predominance of positive RF and ACPA, and significant structural damage and functional disability. As a group, approximately half were within the DAS28-defined treatment target of remission or low disease activity. Almost all patients were on csDMARDs, 41.6% were on b/tsDMARDs, and 34.9% were on oral glucocorticoids at the time of the study.

**Figure 1:**
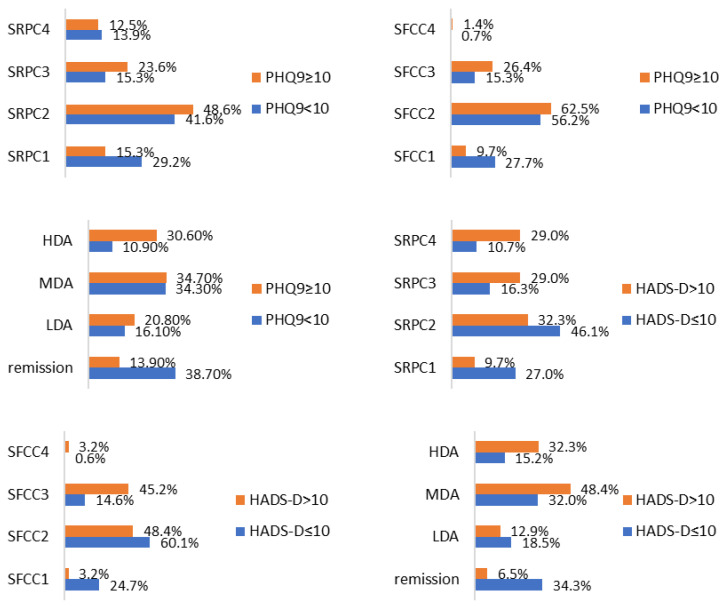

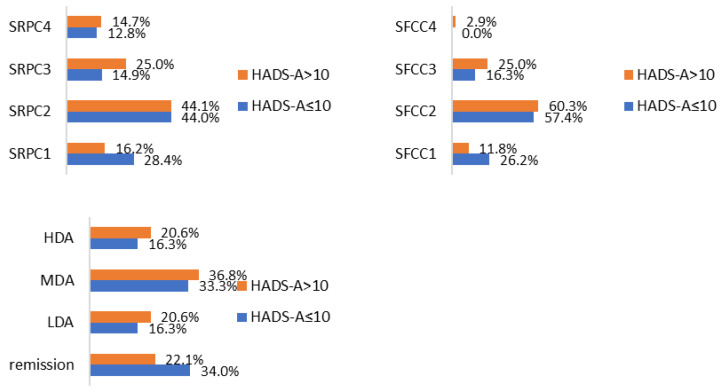
From left to right, Steinbrocker radiographic progression class (SRPC), Steinbrocker functional capacity class (SFCC), and DAS28-defined disease activity (HDA—high, MDA—moderate, LDA—low) according to PHQ9 (*p* = 0.106, 0.014, 0.000), HADS-D (*p* = 0.004, 0.000, 0.003), and HADS-A (*p* = 0.139, 0.015, 0.349).

Medical history revealed a significant proportion of patients with known depression (10.0%) and anxiety (8.1%), and only 7.7% of patients were receiving prescribed antidepressants. Likely depression diagnoses were screened for 34.4% who had a PHQ9 of 10 or more and for 14.8% of patients who had a HADS-D of more than 10 (Table 2). Also, likely anxiety diagnoses were screened for 32.5% who had a HADS-A of more than 10 (Table 2). Compared to patients with a PHQ9 score of below 10, those with a PHQ9 score of 10 or more presented significantly higher numbers of tender and swollen joints, global patient evaluations, DAS28, glucocorticoid treatment frequency, functional stages, and disease activity classes (Table 3, Figure 1). Similarly, compared to patients with a HADS-D score of 10 or below, those with a HADS-D score of more than 10 presented significantly higher mean disease duration, ESR levels, numbers of tender and swollen joints, global patient evaluations, DAS28, radiographic and functional stages, and disease activity classes (Table 3, Figure 1). Compared with patients scoring 10 or below on the HADS-A, those with questionnaire-defined anxiety were more frequently women and they had significantly higher functional staging of RA and higher tender joint counts (Table 3, Figure 1).

PHQ9 and HADS-A scores were significantly higher in women and in patients with more severe joint damage, functional stage, and disease activity class (Table 4). Additionally, HADS-D scores were significantly higher in the same patients and in those with glucocorticoid use.

## 4. Discussion

The likely depression and anxiety diagnoses screened with the self-reported questionnaires PHQ9 and HADS were higher than their known medical history of depression and anxiety. As previously discussed, the reported prevalence of depression and anxiety in RA patients has a significant variability because of the multiple thresholds of the questionnaires used in different studies. For example, when using the HADS measure, the range of estimates for depression was between 14.8% and 48% due to the use of variable cut-off scores. Screening for anxiety utilizing the HADS scale can generate a prevalence that varies from 14.8% to 48% [23].

When evaluating depression or anxiety prevalence in RA, all screening tools need clinical confirmation. Nevertheless, the high difference between the proportion of patients with confirmed diagnosis of depression and anxiety and the possible diagnoses after applying screening questionnaires should raise awareness over the fact that most RA patients with psychiatric disorders are left undiagnosed and untreated, which leads to poor long-term outcomes.

It has been shown in multiple studies that the severity of depression correlates with RA disease activity and with long-term poor outcome [11]. Different observational studies revealed that high depression scores were associated with RA disease activity, without a clear confirmation of causality or direction [24]. A bidirectional relationship between depression and disease activity is possible, as studies in early RA reported that higher joint count and disease activity at baseline are associated with the presence and persistence of depression at 6 months [25]. A study by Fragoulis et al. that evaluated depression and anxiety with the HADS found a significant correlation between the HADS scores for depression and anxiety and DAS28-CRP at 6 and 12 months [26]. Similar results were obtained by Matcham et al., in which persistent depressive and anxiety symptoms were associated with higher values of DAS28 [10]. A systematic review and meta-analysis by Machin et al. that included five studies mentioned a significant correlation between anxiety and increased DAS28 and decreased life quality [27]. The specialized literature has shown that depression leads to higher disease activity through association with the subjective components of the disease activity score (DAS28), such as tender joint count or patient global assessment [28]. Studies by Michelsen et al. and Matcham et al. found a correlation between the presence of depression and anxiety with higher TJC and higher VAS [29,30]. An additional study published by Matcham et al. observed that the presence of depression had a strong correlation with higher DAS28, higher tender joint count, but also higher swollen joint count [10]. Most of the studies suggest that anxiety is significantly associated with the more subjective components of disease activity such as pain levels, patient global assessments, and tender joint count, without any correlations with the swollen joint count or acute phase reactants [27]. Objective measures, such as swollen joint counts [31] and inflammatory markers of disease activity, have been variably associated with baseline depression in different studies. Some studies support the concept of a close relationship between inflammation and depression, as inflammation markers (ESR and CRP) seem to be higher in patients with RA and depression. In the study conducted by Fragoulis et al., CRP levels were correlated with depression scores at baseline and the subsequent follow-up time-points. Conversely, higher CRP levels at baseline have also been shown to distinguish between RA patients who would go on to develop depression. ESR values correlated with the presence of depression at baseline and at the 12 month follow-up [29]. Kekow et al. reported, almost 10 years earlier, higher CRP levels at baseline in patients who had depression compared to those who did not [13]. Overall, our results highlight either a common pathogenic mechanism of RA and depression via pro-inflammatory cytokines or an effect of depression on disease activity and management. Regarding the association between anxiety and CRP levels, there have not been any reported correlations.

Patients with depression also tend to have poor functional status, partly because of poor radiological outcomes in the presence of depression [32]. On the other hand, any improvement in depressive symptoms may lead to improvement in the functional status of the patient [26]. In a cohort of patients with RA and undifferentiated arthritis, Kronisch et al. revealed that the baseline predictors of disability at 1 year are dominantly psychosocial and include high baseline disability, work disability, depression, anxiety, and being overweight [33].

A prospective multicenter observational study by Michelsen et al. found that depression and anxiety were both negative predictors for RA remission after 3 and 6 months of treatment [29], while a pooled analysis of five randomized controlled trials observed that only comorbid depression and not anxiety was associated less frequently with remission [28].

In this study, patients who screened positive for depression according to the utilized threshold (PHQ9 of 10 or more) had significantly higher disease activity class and DAS28, corresponding with the fact that depression correlates with RA disease activity [10]. As the specialized literature mentions, this subgroup of patients also had higher values of the subjective components of disease activity, such as tender joint count and patient global assessment [27]. Additionally, swollen joint count also had high levels [31], which was also reported in other findings. The patients with a PHQ9 of 10 or more had worse functional stages and an increased frequency of glucocorticoid treatment use. Patients with a possible depression diagnosis based on a HADS-D score of more than 10 presented almost similar characteristics to those with a PHQ9 of 10 or more, with some notable differences. They shared higher number of tender and swollen joints, patient global assessment, DAS28, disease activity classes, and functional stages; additionally, they had higher radiographical damage, higher levels of ESR, and significantly higher mean disease duration. A prospective study conducted by Kwiatkowska et al. in 2018 identified disease duration as an important factor in the prevalence of depression [34]. Patients with a HADS-A score of more than 10 were more frequently women, women being more frequently affected by anxiety, and they had higher tender joint counts as other studies suggested [27] and significantly worse functional staging of RA. PHQ9, HADS-D, and HADS-A scores were significantly higher in patients with higher disease activity class, more severe radiographic damage, and greater functional stage. Additionally, PHQ9 and HADS-A had higher values in women, and HADS-D scores were significantly higher in patients with glucocorticoid use.

The results of the current study should be interpreted taking into consideration the following limitations: cross-sectional design (which cannot establish causation or temporal relationships between RA and depression/anxiety); measurement bias of self-reported questionnaire data and possible local culture variations (underreporting symptoms caused by fear of stigmatization or patient–physician relationship influence); the diagnostic tool’s limitation (e.g., clinical interview versus standardized questionnaire); additional unaddressed confounding issues (such as fibromyalgia, chronic pain, side effects and tolerability of RA drugs) and unmeasured variables (socioeconomic status, social support, coping mechanisms, and lifestyle factors); possible selection bias caused by recruitment from a specialized hospital.

## 5. Conclusions

Depression and anxiety are highly prevalent in this RA population and are associated with higher levels of pain, physical disability, and disease activity. Taking into consideration the major impact of these psychiatric diseases on RA patients, it is highly important to screen patients on a routine basis for early referral to a psychiatry specialist for diagnosis and treatment in the hopes of improving the disease activity and life quality of RA patients. 

## Figures and Tables

**Table 1:** General characteristics of RA patients (*n* = 209).

women	92.3%	CRP (mg/L) median (IQR)	4.7 (9.3)
age (y) mean (SD)	62.1 (11.5)	ESR (mm/h) mean (SD)	28.5 (20.1)
RA duration (y) mean (SD)	11.1 (9.9)	TJC28 median (IQR)	3 (5)
RF positive	70.3%	SJC28 median (IQR)	1 (3)
ACPA positive	75.6%	PtGA (mm) mean (SD)	42 (25)
RF and ACPA positive	64.6%	DAS28 * mean (SD)	3.5 (1.5)
SRPC1	24.4%	DAS28 remission	30.1%
SRPC2	44.0%	DAS28 LDA	17.7%
SRPC3	18.2%	DAS28 MDA	34.4%
SRPC4	13.4%	DAS28 HDA	17.7%
SFCC1	21.5%	≥1 csDMARDs	90.9%
SFCC2	58.4%	b/tsDMARD	41.6%
SFCC3	19.1%	glucocorticoids	34.9%
SFCC4	1.0%		

Notes: * DAS28 is calculated with four variables and CRP. Normal ranges: CRP < 5 mg/L; ESR < 20 mm/h. Abbreviations: ACPA—anti-citrullinated protein antibodies; b/cs/tsDMARDs—biologic, conventional synthetic or targeted synthetic disease-modifying antirheumatic drugs; CRP—C-reactive protein; DAS28—disease activity score with 28 joints; ESR—erythrocyte sedimentation rate; HDA—high disease activity; IQR—interquartile range; LDA—low disease activity; MDA—moderate disease activity; PtGA—patient global assessment; RA—rheumatoid arthritis; RF—rheumatoid factor; SD—standard deviation; SFCC—Steinbrocker functional capacity class; SJC—swollen joint count; SRPC—Steinbrocker radiographic progression class; TJC—tender joint count; y—years.

**Table 2:** Depression and anxiety characteristics (*n* = 209).

depression history	10.0%	HADS-D mean (SD)	6.3 (4.0)
anxiety history	8.1%	HADS-D > 10	14.8%
antidepressants	7.7%	HADS-D normal	64.6%
PHQ9 mean (SD)	8.1 (4.9)	HADS-D borderline	21.1%
PHQ9 ≥ 10	34.4%	HADS-D abnormal	14.4%
PHQ9-DS: none/minimal	27.3%	HADS-A	8.6 (4.4)
PHQ9-DS: mild	38.3%	HADS-A > 10	32.5%
PHQ9-DS: moderate	25.4%	HADS-A normal	40.2%
PHQ9-DS: moderate-severe	8.1%	HADS-A borderline	26.8%
PHQ9-DS: severe	0.5%	HADS-A abnormal	33.0%

Abbreviations: DS—depression severity; HADS D/A—Hospital Anxiety and Depression Scale Depression/Anxiety; PHQ-9—Patient Health Questionnaire-9; RA—rheumatoid arthritis; SD—standard deviation.

**Table 3:** Comparison of RA variables according to PHQ9, HADS-D and HADS-A cutoffs.

	PHQ9<10 (*n* = 137)	PHQ9≥10 (*n* = 72)	*p*	HADS-D ≤10 (*n* = 178)	HADS-D >10 (*n* = 31)	*p*	HADS-A ≤10 (*n* = 141)	HADS-A >10 (*n* = 68)	*p*
women	90.5%	95.8%	0.169	91.6%	96.8%	0.315	88.7%	100%	0.004
age (y) mean (SD)	61.5 (11.8)	61.9 (11.0)	0.646	61.2 (11.6)	63.7 (11.0)	0.339	61.0 (11.7)	62.8 (11.2)	0.289
RA duration (y) mean (SD)	10.5 (8.9)	11.5 (10.4)	0.418	10.0 (8.4)	15.2 (13.1)	0.028	10.3 (8.6)	11.9 (10.9)	0.157
RF +	71.3%	70.4%	0.892	70.5%	74.2%	0.672	70.5%	72.1%	0.817
ACPA +	75.0%	78.9%	0.534	75.0%	83.9%	0.284	75.5%	77.9%	0.703
RF, ACPA +	62.8%	68.1%	0.448	62.9%	74.2%	0.226	63.1%	67.6%	0.521
CRP (mg/L) median (IQR)	4.4 (8.1)	6.2 (14.1)	0.094	4.6 (9.0)	5.6 (21.8)	0.119	4.3 (8.5)	6.3 (11.7)	0.183
ESR (mm/h) mean (SD)	26.3 (18.9)	31.9 (20.5)	0.112	26.3 (22.5)	38.7 (22.9)	0.012	26.6 (18.6)	31.4 (21.1)	0.085
TJC median (IQR)	2 (6)	4 (7)	0.000	2 (6)	6 (6)	0.000	2 (6)	4 (6)	0.025
SJC median (IQR)	0 (3)	2 (4)	0.006	0 (3)	3 (5)	0.006	0 (3)	1 (4)	0.216
PtGA (mm) mean (SD)	36 (24)	54 (24)	0.000	40 (26)	57 (19)	0.001	40 (26)	46 (24)	0.169
DAS28 * mean (SD)	3.2 (1.4)	4.1 (1.4)	0.000	3.4 (1.4)	4.5 (1.3)	0.000	3.4 (1.5)	3.8 (1.4)	0.130
≥1 csDMARDs	89.8%	93.1%	0.631	90.4%	93.5%	0.815	92.2%	88.2%	0.607
b/tsDMARD	43.1%	38.9%	0.560	39.9%	51.6%	0.222	41.1%	42.6%	0.835
glucocorticoids	29.9%	44.4%	0.036	33.7%	41.9%	0.375	31.9%	41.2%	0.188

Notes: * DAS28 is calculated with four variables and CRP. Age, disease duration, ESR, PtGA, and DAS28 are tested with two-sided independent t tests, while CRP, TJC, and SJC are tested with Mann–Whitney tests. Nominal variables are tested with χ^2^ tests. Abbreviations: + positive; ACPA—anti-citrullinated protein antibodies; b/cs/tsDMARDs—biologic, conventional synthetic or targeted synthetic disease-modifying antirheumatic drugs; CRP—C-reactive protein; DAS28—disease activity score with 28 joints; ESR—erythrocyte sedimentation rate; IQR—interquartile range; PHQ-9—Patient Health Questionnaire-9; PtGA—patient global assessment; RA—rheumatoid arthritis; RF—rheumatoid factor; SD—standard deviation; SJC—swollen joint count; TJC—tender joint count; y—years.

**Table 4:** Questionnaire scores according to RA characteristics.

		PHQ9	HADS-D	HADS-A
sex	women	8.3 (4.9)	6.3 (4.0)	8.9 (4.5)
	men	5.2 (3.5)	5.6 (3.8)	5.5 (2.8)
	*p*	0.013	0.466	0.003
RF	negative	8.1 (4.3)	5.9 (3.7)	8.6 (4.0)
	positive	8.1 (5.1)	6.4 (4.1)	8.7 (4.6)
	*p*	0.973	0.419	0.888
ACPA	negative	7.6 (4.4)	5.9 (3.6)	8.5 (4.5)
	positive	8.2 (5.0)	6.4 (4.1)	8.7 (4.4)
	*p*	0.461	0.434	0.789
Steinbrocker radiographic stage	1	5 (5)	4 (4)	7 (6)
	2	8 (8)	6 (4)	9 (8)
	3	9 (6)	8 (7)	10 (7)
	4	7 (6)	8 (7)	8 (8)
	*p*	0.011	0.009	0.045
Steinbrocker functional stage	1	5 (5)	4 (5)	6 (6)
	2	7 (8)	6 (6)	9 (7)
	3	9 (7)	9 (6)	10 (8)
	4	11 (5)	9 (7)	17 (4)
	*p*	0.001	0.000	0.005
RA disease activity *	remission	6 (5)	4 (5)	7 (7)
	low	8 (9)	5 (5)	8 (8)
	moderate	8 (7)	6 (6)	9 (6)
	high	10 (9)	9 (6)	10 (6)
	*p*	0.000	0.000	0.058
b/tsDMARD	no	8.3 (4.8)	6.2 (3.8)	8.5 (4.3)
	yes	7.8 (4.9)	6.3 (4.2)	8.8 (4.6)
	*p*	0.339	0.929	0.619
glucocorticoids	no	7.8 (5.1)	5.8 (3.9)	8.2 (4.5)
	yes	8.6 (4.4)	6.2 (4.0)	9.4 (4.3)
	*p*	0.175	0.018	0.074

Notes: * According to DAS28 with four variables and CRP. Differences between PHQ9 and HADS were assessed with two-sided independent t tests for sex, RF status, ACPA status, b/tsDMARD treatment, and glucocorticoid treatment and are reported as mean (SD), and with Kruskal–Wallis tests for SRS stages, SFR stages, and RA activity classes and are reported as median (IQR). Abbreviations: ACPA—anti-citrullinated protein antibodies; b/tsDMARDs—biologic or targeted synthetic disease-modifying antirheumatic drugs; CRP—C-reactive protein; DAS28—disease activity score with 28 joints; HADS D/A—Hospital Anxiety and Depression Scale Depression/Anxiety; IQR—interquartile range; PHQ-9—Patient Health Questionnaire-9; RA—rheumatoid arthritis; RF—rheumatoid factor; SD—standard deviation.

## Data Availability

The data presented in this study are available on request from the corresponding author due to patient confidentiality.

## Abbreviations

The following abbreviations are used in this manuscript:

RA	Rheumatoid arthritis
HADS	Hospital Anxiety and Depression Scale
PHQ9	Patient Health Questionnaire
HADS-D	Hospital Anxiety and Depression Scale—Depression subscale
HADS-A	Hospital Anxiety and Depression Scale—Anxiety subscale
DAS28	Disease Activity Score—28 joints count
VAS	Visual analogue scale
ESR	Erythrocyte sedimentation rate
CRP	C reactive protein
DSM	Diagnostic and Statistical Manual of Mental Disorders
ICD	International Classification of Diseases
IDD	Inventory to Diagnose Depression
CESD	Center for Epidemiological Studies Depression Scale
BDI	Beck Depression Inventory
GDS	Geriatric Depression Scale
cs/b/ts DMARD	Conventional synthetics/biologics/targeted synthetics Disease-modifying antirheumatic drugs
ACPA	Anti-citrullinated protein antibodies
TJC	Tender joint count
SJC	Swollen joint count
SRPC	Steinbrocker radiographic progression
SFCC	Steinbrocker functional capacity class
IQR	Interquartile range
SD	Standard deviation
PtGA	Patient global assessment
